# Methionine-restricted diet inhibits growth of MCF10AT1-derived mammary tumors by increasing cell cycle inhibitors in athymic nude mice

**DOI:** 10.1186/s12885-016-2367-1

**Published:** 2016-06-03

**Authors:** J. R. Hens, I. Sinha, F. Perodin, T. Cooper, R. Sinha, J. Plummer, C. E. Perrone, D. Orentreich

**Affiliations:** Orentreich Foundation for the Advancement of Science, Inc., 855 Route 301, Cold Spring, NY 10516 USA; Biochemistry and Molecular Biology, Penn State College of Medicine, 500 University Drive, Hershey, PA 17033 USA; Comparative Medicine, Penn State College of Medicine, 500 University Drive, Hershey, PA 17033 USA

**Keywords:** Methionine, Diet, P21, Breast cancer, Cell cycle, Inhibitors, CDKN1a

## Abstract

**Background:**

Dietary methionine restriction (MR) improves healthspan in part by reducing adiposity and by increasing insulin sensitivity in rodent models. The purpose of this study was to determine whether MR inhibits tumor progression in breast cancer xenograft model and breast cancer cell lines.

**Methods:**

Athymic nude mice were injected with MCF10AT1 cells in Matrigel® and fed a diet containing either 0.86 % methionine (control fed, CF), or 0.12 % methionine (MR) for 12 weeks. Plasma amino acid concentrations were measured by UPLC, and proliferation and apoptosis were examined using RT-PCR, immunohistochemistry, and Cell Titer 96® Aqueous One Solution Cell Proliferation assay.

**Results:**

Mice on the MR diet had reduced body weight and decreased adiposity. They also had smaller tumors when compared to the mice bearing tumors on the CF diet. Plasma concentrations of the sulfur amino acids (methionine, cysteine, and taurine) were reduced, whereas ornithine, serine, and glutamate acid were increased in mice on the MR diet. MR mice exhibited decreased proliferation and increased apoptosis in cells that comprise the mammary glands and tumors of mice. Elevated expression of P21 occurred in both MCF10AT1-derived tumor tissue and endogenously in mammary gland tissue of MR mice. Breast cancer cell lines MCF10A and MDA-MB-231 grown in methionine-restricted cysteine-depleted media for 24 h also up-regulated P21 and P27 gene expression, and MDA-MB-231 cells had decreased proliferation.

**Conclusion:**

MR hinders cancer progression by increasing cell cycle inhibitors that halt cell cycle progression. The application of MR in a clinical setting may provide a delay in the progression of cancer, which would provide more time for conventional cancer therapies to be effective.

**Electronic supplementary material:**

The online version of this article (doi:10.1186/s12885-016-2367-1) contains supplementary material, which is available to authorized users.

## Background

One of the most common cancers worldwide is breast cancer, and it is the second leading cause of mortality in women from the United States [1, 2]. Although conventional therapies and surgical approaches have been developed, none are completely effective in removing and annihilating the cancer.

Cancer cells alter their metabolic machinery to maintain a high level of metabolism and prevent the depletion of a host of substrates, such as glucose and amino acids that are used for energy. A significant aspect of this reprogramming involves changes in the glycolytic pathway, referred to as the Warburg effect [3, 4]. These changes include an increase in pyruvate that is generated via the glycolytic pathway. Pyruvate is converted to lactic acid instead of acetyl-CoA which enters the TCA cycle and ultimately forms citrate. In addition to metabolic changes from Warburg effect, some cancers depend on glutamate metabolism for fixing ammonia to acquire the nitrogen required for cellular growth [5]. Further, glutamine synthetase is a target for activated β-catenin and is regulated by the oncogene Myc [5], which connects the metabolic regulation of cancer cells to several important growth and developmental signaling pathways.

Dietary nutritional control may be a feasible option for supplementing cancer treatment. The use of caloric restriction (CR) is effective in inhibiting cancer development in non-human primates and rodents [6, 7] and the onset of age-related diseases [6]. However, recent evidence suggests that CR can reduce function of the immune system [8, 9], which may not be ideal for people already fighting cancer. Further, the general population is not in favor of reducing food consumption as required by CR. A viable alternative to CR may be to restrict the intake of dietary methionine.

Methionine is an essential amino acid with a multitude of functions. It is prominent in protein translation, since it is the N-terminal amino acid of most mammalian proteins. Methionine is a precursor of glutathione, a tripeptide that reduces reactive oxygen species (ROS) and protects cells from oxidative stress. Methionine is needed for polyamine synthesis, in which polyamines function during nuclear and cell division. Moreover, methionine is a precursor of *S*-adenosyl methionine the major source of methyl groups needed for methylation of DNA, proteins and low *M*_r_ biomolecules/metabolites. In rodents, a diet low in methionine (20-35 % of regular chow) reduced adiposity in the fat depots and reduced blood levels of lipids, glucose, IGF-1, and leptin, while elevating levels of FGF21 and adiponectin [10–15]. Reduction in mitochondrial free radical production and oxidative stress also occurs during MR in organs, such as, liver, heart, and brain [16–18]. Finally, MR in rodents promotes longevity and delays onset of age-related impairments and chronic diseases [10, 19–21].

In intestinal, colon, and prostate cancer models, methionine levels were restricted or excluded from diets to examine the effects on tumor progression. In an intestinal tumorigenesis model with Apc(Min/+) mice, folate deficiency in combination with depletion of choline, methionine, and vitamin B12 resulted in reduced tumor size in mice treated by 5 weeks of age, but produced no difference when started at 10 weeks of age [22]. Azoxymethane-treated rats when fed MR diet had a significant reduction in formation of aberrant crypt foci in colon, suggesting an inhibition of cell proliferation [21]. More recently, dietary MR in TRAMP mouse model demonstrated a decrease in prostatic intraepithelial neoplasia with a concomitant drop in plasma IGF-1 levels and reduced proliferation in prostate lobe-specific manner [23]. In human prostate cancer cell lines grown in the absence of methionine, the expression of cell cycle inhibitors P21 and P27 increased and were identified as a possible mechanism for halting the cell cycle and increasing apoptosis [24]. These studies suggest that methionine depletion in human prostate cells can inhibit proliferation either by halting the cell cycle at the G1/S checkpoint or by directing cells to go through apoptosis. However, little is known about how MR alters the cell cycle in other cancers, such as breast cancer. The present study uses a xenograft model for breast cancer, an immortalized human breast cell line, and an invasive breast cancer cell line to examine whether MR alters cell cycle inhibitors that could inhibit tumor progression.

## Methods

### Xenograft model for testing efficacy of methionine restriction

MCF10AT1, human transformed breast cells, were a gift obtained from Steven Santner at Barbara Ann Karmanos Cancer Institute, Detroit, MI. The cells were maintained in DMEM/F12 (Invitrogen, Carlsbad, CA) with 5 % horse serum, 0.029 M sodium bicarbonate, and 10 mM HEPES, and supplemented with insulin (10 μg/ml), EGF (20 ng/ml), hydrocortisone (0.5 μg/ml), cholera toxin (100 ng/ml), and 1 % penicillin-streptomycin solution. Cells were routinely passaged weekly and maintained in 5 % CO_2_ at 37 °C.

The procedures and treatments used on the athymic nude mice were reviewed and approved by Penn State College of Medicine Institutional Animal Care and Use Committee (IACUC#2012-082), and followed standard procedures described in *Guide for the Care and Use of Laboratory Animals*, 8^th^ edition from the National Research Council. A completed ARRIVE guidelines checklist is included in Additional file 1. A total of ten million MCF10AT1 cells with matrigel (1:1 (v/v), BD Biosciences, San Jose, CA) were injected subcutaneously into the left flank region near the mammary fat pad of each of 40 female athymic nude mice (Strain-088 homozygous; 8 weeks of age; Charles River, Wilmington, MA). The mice were fed sterile standard rodent diet for 1 week after cell implantation. These mice were divided into 2 feeding groups: (a) 20 mice on a sterile, a chemically-defined AIN-76-based diet containing 0.86 % methionine (control-fed diet; CF); and (b) 20 mice on a sterile chemically-defined AIN-76-based diet containing 0.12 % methionine (methionine-restricted diet; MR). The mice were housed in groups of five and maintained on a 12 h light-dark cycle and fed these diets ad libitum for 12 weeks. Body weights and average diet intake for each group were measured weekly.

At the end of the study, unfasted mice were euthanized using CO_2_; whole blood was collected by cardiac puncture, centrifuged, and plasma was collected and stored at −80 °C for analysis. Plasma samples were analyzed for amino acid content, insulin, IGF-1, FGF21, leptin, adiponectin, cholesterol, triglycerides, and glucose.

Tumors were excised, weighed, and measured prior to fixing in 10 % neutral buffered formalin (NBF). Tumor volumes were determined using the formula: 0.523 x *a*^2^ x *b*, where *a* is the smallest diameter and *b* is the largest. The fixed tumors were paraffin-embedded, sectioned and stained for hematoxylin & eosin. Histopathology analysis was performed by Dr Timothy Cooper (Penn State College of Medicine), and the percent of lesions morphologically resembling mild, moderate, or florid intraductal papillomas (IDPs); ductal carcinoma *in situ* (DCIS); or invasive carcinoma were calculated. The tumor sections also were used for immunohistochemical analysis of proliferation and apoptosis for all groups. Additionally, the mammary glands from 10 mice of each group were fixed in NBF for histology. At sacrifice, organ weights for liver, kidneys, spleen, mammary fat pads, and left gastrocnemius muscle were recorded.

### Tumor cell proliferation and apoptosis

The percentages of Ki67 and cleaved caspase-3 positive cells counted in triplicate under a high power field (400X) were used as a measure of proliferation and apoptosis, respectively. Briefly, formalin-fixed paraffin-embedded mammary and MCF10AT1 tumor sections were hydrated, subjected to antigen retrieval, and incubated at room temperature with 1:100 anti-Ki67 antibody (M7240, Dako North America Inc, Carpinteria, CA) for 30 min and 1:100 cleaved-caspase-3 antibody (9661, Cell Signaling, Danvers, MA) for 1 h. Slides were developed using the Dako Envision™ +/HRP Polymer detection system (K4001, Dako North America Inc, Carpinteria, CA) and visualized with 3,3'-Diaminobenzidine (DAB) chromagen followed by hematoxylin counterstain. Staining was completed using a Dako Autostainer Plus.

### Amino acid analysis

Plasma amino acid concentrations were measured using the Acquity UPLC (Waters Corporation, Milford, MA) with the AccQ.Tag Ultra derivatization Kit (Waters Corporation, Milford, MA). Plasma samples were deproteinized with a solution of 10 % sulfosalicylic acid and 250 pmol/μl of norvaline was used as the internal standard.

### Assays on plasma parameters

Enzyme-linked immunosorbent assay (ELISA) kits were used to measure insulin (ALPCO Diagnostics, Salem, NH), leptin, IGF-1, adiponectin (R&D Systems, Minneapolis, MN), and FGF21 (Millipore Corp., Billerica, MA). Colorimetric assays were used to determine plasma triglycerides (TG) and total cholesterol (TC) (Thermo Electron Corp. Beverly, MA). Blood glucose was measured using an AbbottH Freestyle glucometer and glucose test strips.

### Methionine restriction in cell culture

MDA-MB-231 and MCF10A cells were obtained from American Type Culture Collection (Manassas, VA). MDA-MB-231 cells were maintained in DMEM/F12 media with 10 % fetal bovine serum, and MCF10A cells were maintained in DMEM/F12 media containing 5 % horse serum with the addition of EGF (20 ng/ml), hydrocortisone (0.5 μg/ml), cholera toxin (100 ng/ml), and insulin (10 μg/ml). Cells were routinely passaged weekly and maintained in 5 % CO_2_ at 37 °C. Methionine restricted media were specially formulated without methionine, cysteine, and cystine (Life Technologies, Grand Island, NY), and the FBS or horse serum was dialyzed in PBS pH 7.2 three times over a period of 24 h at 4 °C using a Slide-A-Lyzer™ dialysis flask with a 3.5 K MWCO (Thermo Fisher Scientific, Waltham, MA) to remove all amino acids. Media were specially formulated to be 80 % reduced in methionine from the regular DMEM/F12 media (17.24 mg Met/L), with no cysteine or cystine, but with all amino acids and non-essential amino acids (methionine-restricted, cysteine-depleted (MRCD) media). Cells were plated and grown to 80 % confluence, washed once with PBS, and then given either MRCD or regular DMEM/F12 media. Twenty-four hours later, RNA was harvested from cells using Tri-Reagent (Molecular Research Center, Cincinnati, OH), according to the manufacturer’s instructions.

### In vitro proliferation assay

Cells (MCF10A and MDA-MB-231) were plated with 40 μl of 1X10^5^ cells/ml per well in a 96 well cell-culture treated dish. The next day cells were washed with PBS, and then treated with either MRCD or regular DMEM/F12 media. At 0 and 24 h cells were assayed for changes in proliferation by treating with the Cell Titer 96® Aqueous One Solution Cell Proliferation assay reagent, according to the manufacturer’s instructions (Promega, Madison, WI). Four experiments with 6 replicates per treatment were conducted. Results were analyzed using a 2-way ANOVA with Sidak’s comparison test.

### Real-time PCR

RNA isolated from frozen tissue or cells from culture was converted to cDNA using the Verso cDNA synthesis kit (Thermo Fisher Scientific, Waltham, MA) according to the manufacturer’s instructions. Primers were generated for HPRT, P21, P27, and cyclin D1 (Additional file 1: Table S1). Real-time PCR was performed using a SYBR-green GoTaq qPCR system (Promega, Madison WI), with 40 cycles at 95 °C for 15 s, and then 60 °C for 1 min using Applied Biosystems StepOne Plus Real-Time PCR System. Ct values were analyzed using the ΔΔ − Ct method [25].

### RNA isolation and RT-PCR from paraffin sections

Ten micron sections were obtained from the formalin-fixed, paraffin-embedded MCF10AT1 tumors and mammary fat pads of the athymic nude mice. Five sections were collected in a microcentrifuge tube, and RNA was isolated using miRCURY RNA isolation kit (Exiqon, Woburn, MA) according to the manufacturer’s instructions. RNA (250 ng) was converted into cDNA for real-time PCR analysis and analyzed as described above.

### The Cancer Genome Atlas data analysis

The cBioPortal for Cancer Genomics (http://www.cbioportal.org) was used to assess the changes in P21 and P27 expression in breast cancer patients from the Cancer Genome Atlas (TCGA) invasive breast carcinoma cancer study (TCGA, Nature 2012) [26, 27]. This data set was screened for those patients containing data on mRNA levels from Agilent microarrays. Data retrieved from the TCGA controlled-access database was collected using tumors from patients who provided informed consent according to guidelines laid out by the TCGA Ethics, Law and Policy Group which is in compliance with the Helsinki Declaration (http://www.wma.net/en/30publications/10policies/b3/index.html).

### Statistical analysis and data presentation

Histological analyses of mammary and tumor sections from the nude mice were analyzed using an unpaired Mann-Whitney one-tailed t-test to determine significance. Ct values from RT-PCR results were analyzed unpaired one-tailed t-test to determine significance. In vitro proliferation assay data were analyzed using a 2-way ANOVA with Sidak’s comparison test. All analyses were performed using the statistical software package GraphPad PRISM (GraphPad Software Inc, La Jolla, CA).

## Results

### Physiological changes

Physiological parameters of mice on CF and MR were examined. Similar to previous studies [11], MR reduced the body weight of mice, whereas percent weight of liver, spleen, kidney, and mammary fat pad (MFP) were not changed (Table 1). Muscle mass decreased in the mice on MR diet. Future studies may provide a more in depth understanding of the changes that were observed in the muscle.

**Table 1:** Physiological parameters of mice on MR and CF diets. A 2-tailed t-test was conducted

	CF			MR		
	Mean (g) SD	Mean (%) SD	N	Mean SD	Mean (%) SD	N
Body weight	26.6 ±2.79		18	23.9 ±2.3***		20
Liver	1.13 ±0.15	4.35 ±0.43	19	0.988 ±0.13 **	4.15 ±0.49	20
Spleen	0.102 ±0.02	0.383 ±0.07	19	0.104 ±0.15	0.436 ±0.59	20
MFP (both)	0.271 ±0.06	1.01 ±0.20	18	0.275 ±0.10	1.13 ±0.35	20
Kidney (both)	0.343 ±0.04	1.33 ±0.19	19	0.303 ±0.05 **	1.27 ±0.19	20
Muscle-left side	0.123 ±0.02	0.475 ±0.89	19	0.081 ±0.03 ****	0.339 ±0.15 **	20
Tumor-wt (mg)	20.2 ±6.1 ^a^	0.079 ±0.30	15	114 ±4 03 ^a^ ****	0.049 ±0.02***	20

**** *p* ≤ 0.0001, *** *p* ≤ 0.001, ** *p* ≤ 0.01

^a^ tumor is in mg not g

One of the hallmarks of cancer is increased metabolic activity of the cells. Decreased plasma amino acid levels in cancer patients supports the concept that the tumor is parasitizing the organism and altering protein metabolism that would then affect the amino acid concentrations [28]. In this study, increased levels of alanine, glutamine, histidine, ornithine, and serine occurred in the plasma, which may suggest that MR is maintaining or improving the metabolic condition of the animals (Table 2). Equally important is the finding that the sulfur-containing amino acids (methionine, cysteine, and taurine) were reduced significantly in plasma, providing clear evidence that the MR diet reduces sulfur-containing amino acids throughout the animal.

**Table 2:** Plasma amino acid concentrations from mice on MR and CF diets. Levels of methionine, cysteine, and taurine in the plasma from mice on the MR diet are significantly lower than those on the control diet. The Kruskal-Wallis test was used to analyze the differences in amino acid concentration

	CF		MR	
	Mean (nmol/mL) SD	N	Mean (nmol/mL) SD	N
Ala	398 ±85	17	520 ±122 ***	18
Arg	133 ±29	17	117 ±21	18
Asp	11.8 ±4.9	16	10.3 ±3.1	18
Cys	4.39 ±2.4	13	2.22 ±0.44 *	9
Gln	622 ±89	17	812 ±106****	18
Glu	106 ±20	17	117 ±19	18
His	88.8 ±11	17	112 ±20***	18
Ile	94.8 ±32	17	108 ±27	18
Leu	139 ±47	17	180 ±46**	18
Met	248 ±125	17	46 9 ±17****	18
Orn	62.7 ±29	17	114 ±41***	18
Phe	114 ±42	17	132 ±39	18
Pro	69.5 ±8.6	17	82.4 ±34	18
Ser	218 ±34	17	339 ±84****	18
Tau	226 ±50	17	77.1 ±35****	18
Thr	346 ±94	17	383 ±136	18
Tyr	89.5 ±19	17	107 ±28*	18
Trp	130 ±29	17	128 ±29	18

**** *p* ≤ 0.0001, *** *p* ≤ 0.001, ** *p* ≤ 0.01, * *p* ≤ 0.05

**Fig. 1:**
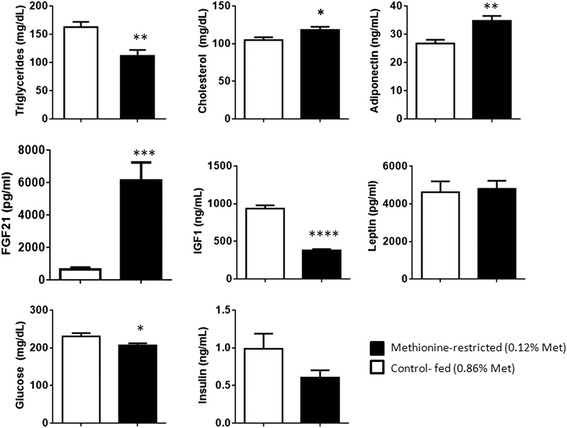
Selected analytes measured in the plasma from nude mice on MR or CF diet. A 2-tailed t-test with Welch’s correction for unequal variance was conducted. **** *p* ≤ 0.0001, *** *p* ≤ 0.001, ** *p* ≤ 0.01, * *p* ≤ 0.05

As predicted from previous studies of mice on a MR diet [11, 29], reductions in plasma triglycerides, IGF1, and glucose were observed (Fig. 1). Plasma levels of adiponectin and FGF21 increased significantly in the MR mice compared to the CF mice (Fig. 1), similar to what was reported previously to occur in rodents on MR [11, 13, 30]. Cholesterol levels were slightly elevated in the MR mice. Leptin was not significantly changed (Fig. 1), whereas leptin levels of C57BL/6 J mice on a MR diet were reported to be lower [11]. These differences in leptin levels may be due to strain differences between the two studies.

### Methionine restriction inhibits tumor progression

**Fig. 2:**
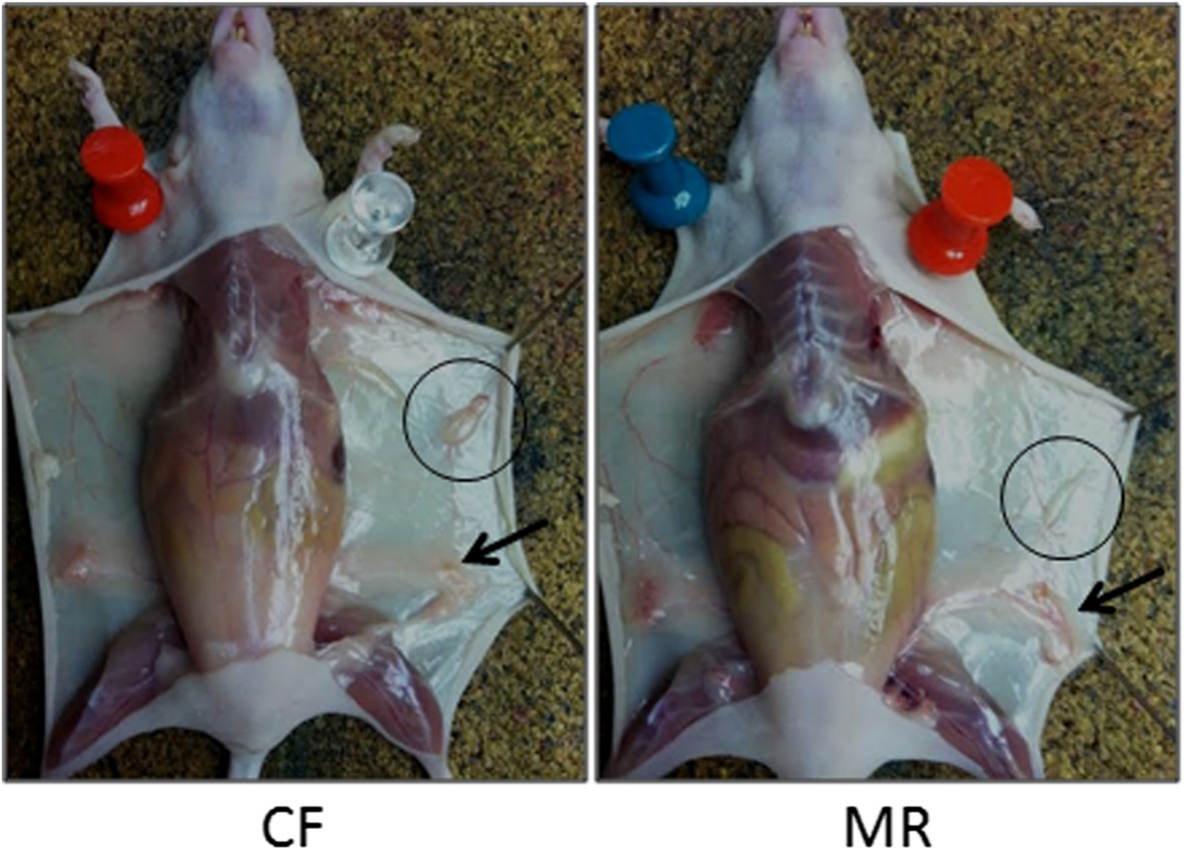
Representative MCF10AT1 breast tumors (black circles left-flank) in CF and MR fed nude mice at termination. Left mammary gland (#4) is indicated with arrows

The MCF10AT1 cell-derived tumors from mice on MR diet were reduced in size when compared to the mice on CF diet (Representative animals from MR and CF groups in Fig. 2). Tumor weights averaged 20.2 ± 6.1 mg in mice on the CF diet. The mice on the MR diet had a notable decrease in tumor weight to 11.4 ± 4.0 mg that corresponds to a reduction of 55 % (*p* ≤ 0.0001) in MR mice when compared to the CF mice (Table 1). Descriptive characteristics of the tumors indicated that the mice on the MR diet trended to have fewer florid IDPs (Table 3). There were no significant differences in mild or moderate IDPs, but there was a significant difference in the number of DCIS lesions between the two groups (Table 3). This suggests that lesions formed prior to the initiation of the MR diet were not eliminated by MR, but that MR slowed tumor progression and reduced the size of tumors formed by the implanted MCF10AT1 cells. The fact that there was a population of mice that acquired an invasive carcinoma phenotype suggests that some cells may be able to escape the MR inhibition through an unknown mechanism.

**Table 3:** Characteristics of tumor lesions formed from MCF10AT1 cell implants in the nude mice. Classifications in order of tumor progression included mild intraductal papilloma (IDP), moderate IDP, florid IDP, ductal carcinoma *in situ* (DCIS), and invasive carcinoma

Lesions	CF		MR	
	Mean (%) SD	*N*	Mean (%) SD	*N*
Mild IDP	46.0 ± 19	15	49.4 ±15	18
Moderate IDP	24.7 ±13	15	26.7 ±11	18
Florid IDP	6.93 ±6.7	15	3.89 ±6	18
DCIS	1.07 ±2.8	15	0 ± 0 *	18
Invasive carcinoma	21.3 ±15	15	20.0 ±13	18

* *p* ≤ 0.05

### Methionine restriction decreases proliferation while increasing apoptosis

**Fig. 3:**
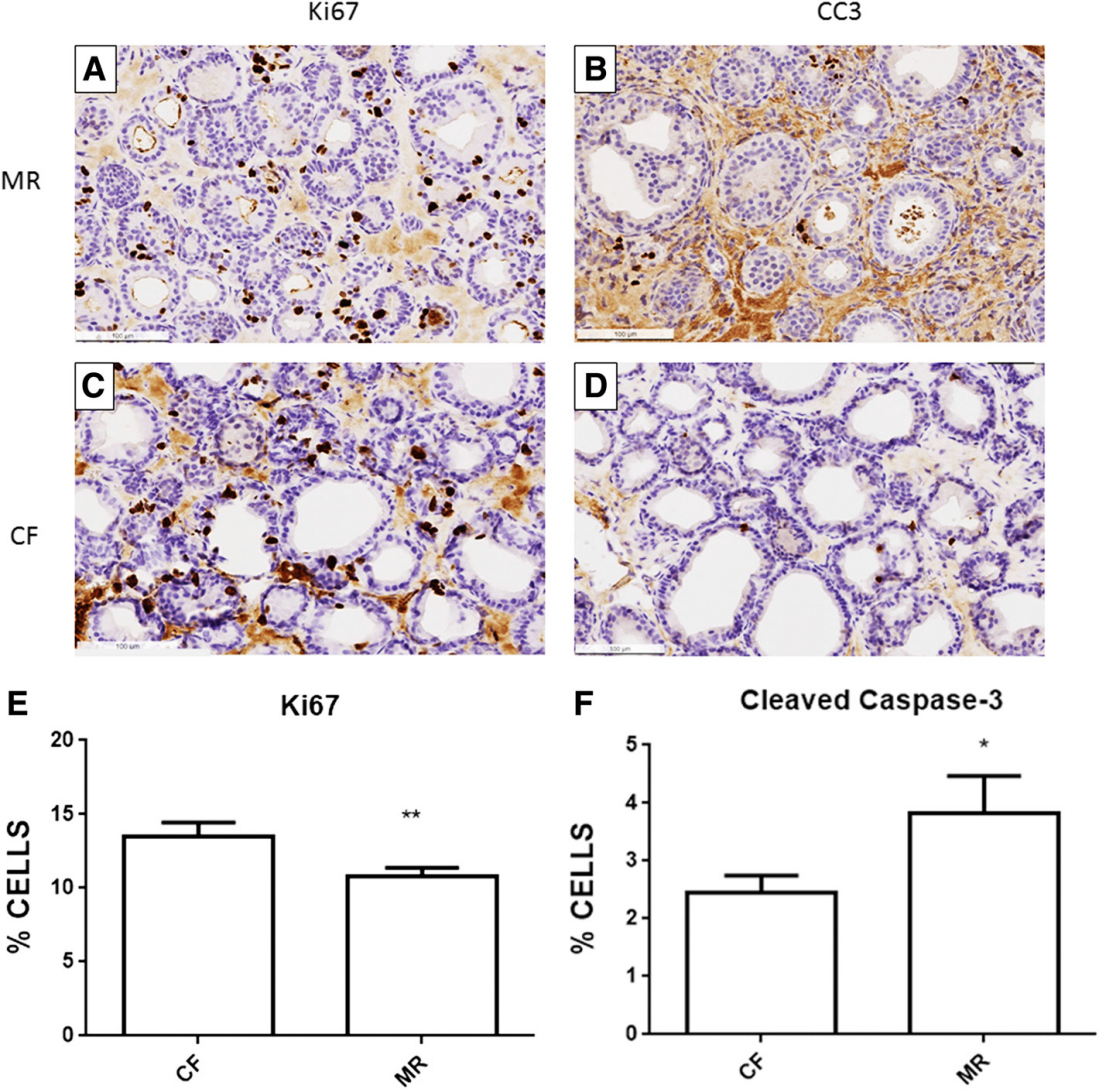
Increases in apoptosis and decreases in proliferation occur in mice on the MR diet when compared to mice on the CF diet. **a**, **c** are representative images of Ki67 positive staining in mammary tissue near the tumor. **b**, **d** represent images of cleaved caspase-3 (CC3) positive staining in the tissue. **a**, **b** are representative sections from mice on the MR diet and **c**, **d** are representative sections from mice on the CF diet. **e** and **f** quantify the Ki67 and cleaved caspase-3 stained tissue sections from 20 animals per treatment, respectively. Mann-Whitney unpaired one-tailed t-test was performed on the samples. * *p* ≤ 0.05, ** *p* ≤ 0.01

To understand a possible mechanism for the decreased tumor size in the mice on the MR diet, proliferation and apoptosis were examined. Histological sections of the both tumor and nearby mammary tissue were stained for Ki67, a marker for proliferation. A total of 20 animals per treatment were examined for levels of Ki67 staining. Representative images demonstrate that the number of Ki67-positive stained cells was reduced in the tissue from mice on the MR diet (Fig. 3a and c). The number of proliferating cells in the tumor tissue was 13.8 ± 0.8 % cells in CF mice, while 11.6 ± 1.1 % cells in MR mice (Fig. 3e; *p* < 0.05). Consecutive sections of tumor tissue were stained for activated caspase-3, as an indicator of apoptosis (Fig. 3b and d); MR mice had significantly elevated levels of activated caspase-3 (MR: 3.82 ± 0.66 % cells, *p* < 0.05), compared with CF mice (CF: 2.44 ± 0.30 % cells) (Fig. 3f).

### Changes in cell cycle regulators by methionine restriction

**Fig. 4:**
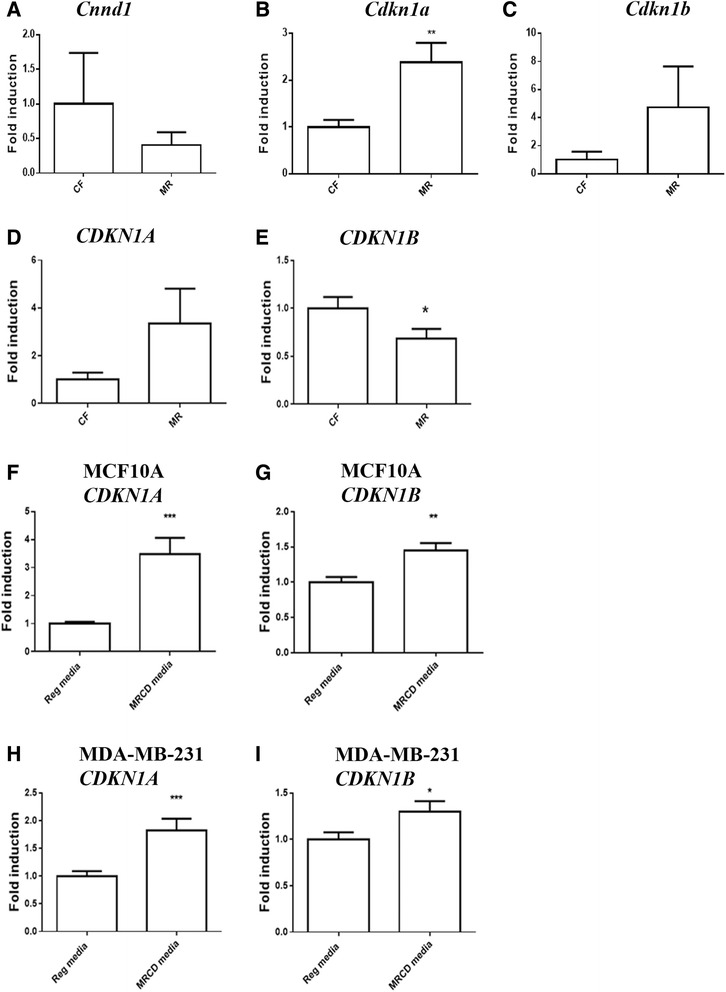
Methionine restriction increases cell cycle inhibitors P21 and P27, while decreasing cyclin D1. **a** Murine cyclin D1 expression from mammary tissue near the MCF10AT1 tumor in CF and MR mice. *N* = 5 CF, =3 MR. **b** Murine P21 expression from mammary tissue sampled near tumor in nude mice on MR or CF diet. *N* = 9 CF, 7 = MR; **c** Expression of murine P27 from mammary tissue sampled near the tumor in nude mice on MR and CF diet. *N* = 7 CF, 7 = MR. **d** Real-time PCR on MCF10AT1 tumor from paraffin sections demonstrated a trend towards an increase in human P21 expression. **e** Real-time PCR on MCF10AT1 tumor from paraffin sections demonstrated a decrease in human P27 expression in the MCF10AT1 cells that comprise the human tumor (*p* = 0.09). D. and E. both had *N* = 5 animals each for CF and MR. **f** Increased P21 expression by real-time PCR in the breast cancer cell line MCF10A after growing cells in in methionine-restricted cysteine-depleted (MRCD) media for 24 h. **g** Increased P27 expression by real-time PCR in the breast cancer cell line MCF10A after growing cells in MRCD media for 24 h. **h** Increased P21 expression by real-time PCR in the breast cancer cell line MDA-MB-231 after growing cells in MRCD media for 24 h. **i** Increased P27 expression by real-time PCR in the breast cancer cell line MDA-MB-231 after growing cells in MRCD media for 24 h. **f**-**i** Cell culture experiments *N* = 4, with 3 replicates in each experiment. Mann-Whitney unpaired one-tailed t-test., ***p ≤ 0.001. ** *p* ≤ 0.01, * *p* ≤ 0.05

The decrease in proliferation combined with the increase in apoptosis suggests that MR may perturb the cell cycle. To investigate this possibility, RNA was harvested from frozen mammary tissue obtained from CF- and MR-treated nude mice. Expression of murine cell cycle inhibitors P21 and P27 and the cell cycle regulator cyclin D1 were measured by real-time PCR in the nude mice. Although there was a slight decrease in cyclin D1, this effect was not significant (Fig. 3a). P21 expression was significantly increased (Fig. 4b, *p* ≤ 0.01) in the mammary gland of MR mice, while P27 was not affected (Fig. 4c). To determine whether the human cell-derived tumors were affected similarly by MR, paraffin sections of embedded tumors from CF or MR mice were collected, and RNA was isolated. Real-time PCR using primers specific to human P21 and P27 were examined. There was a trend indicating that human P21 was increased in the tumors from MR fed mice (Fig. 4d, *p* = 0.09), and a slight decrease in P27 (Fig. 4e, *p* ≤ 0.05).

**Fig. 5:**
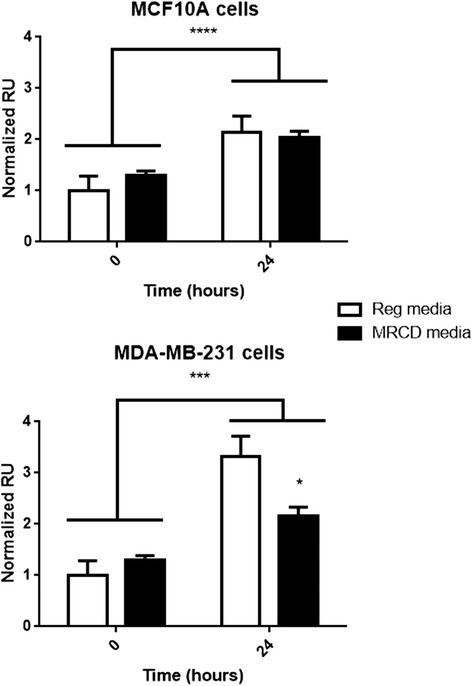
Reduced levels of proliferation were seen by 24 h in MDA-MB-231 and to a lesser degree in MCF10A cells. In both cell types, time had significant affect on proliferation. In MDA-MB-231 cells, time, diet, and the interaction of time and diet on proliferation were significantly affected by MRCD media, whereas in MCF10A cells only an interaction with time and MR affected proliferation. Data was normalized to cells grown in regular media at time 0 and analyzed using a two-way ANOVA with Sidak’s multiple comparisons. ****p ≤ 0.0001, ***p ≤ 0.001, ** *p* ≤ 0.01, * *p* ≤ 0.05

To determine whether the changes in cell cycle control could occur in other breast cancer cells, breast cancer cell line MDA-MB-231 and MCF10A immortal cells were examined under conditions of MR. Cells were grown in regular media and plated at 70 % confluency. The next day, media was changed to either regular media (17.24 mg/L of methionine), or MRCD (3.45 mg/L of methionine) media. Cells were harvested for RNA extraction after 24 h. Real time PCR revealed in both MCF10A and MDA-MB-231 cells that P21 (*p* ≤ 0.001) and P27 (MCF10A *p* ≤ 0.01, MDA-MB-231 *p* ≤ 0.05) were significantly increased by MR (Fig. 4f-i). This suggests that the MR effect on the cell cycle inhibitors may be a similar response to MR in both breast cancer cells and immortalized breast cells. Both MCF10A and MDA-MB-231 cells were examined for changes in proliferation over 24 h (Fig. 5). At 24 h, MDA-MB-231 had a significant reduction of proliferation (*p* ≤ 0.05) by diet, and was further affected by the interaction of diet and time (*p* ≤ 0.001). Proliferation in MCF10A cells was not initially affected by MR, but there were differences in proliferation over time (*p* = 0.0013). The differences in proliferation may indicate that MR may be more effective at hindering invasive breast cancer cells.

**Fig. 6:**
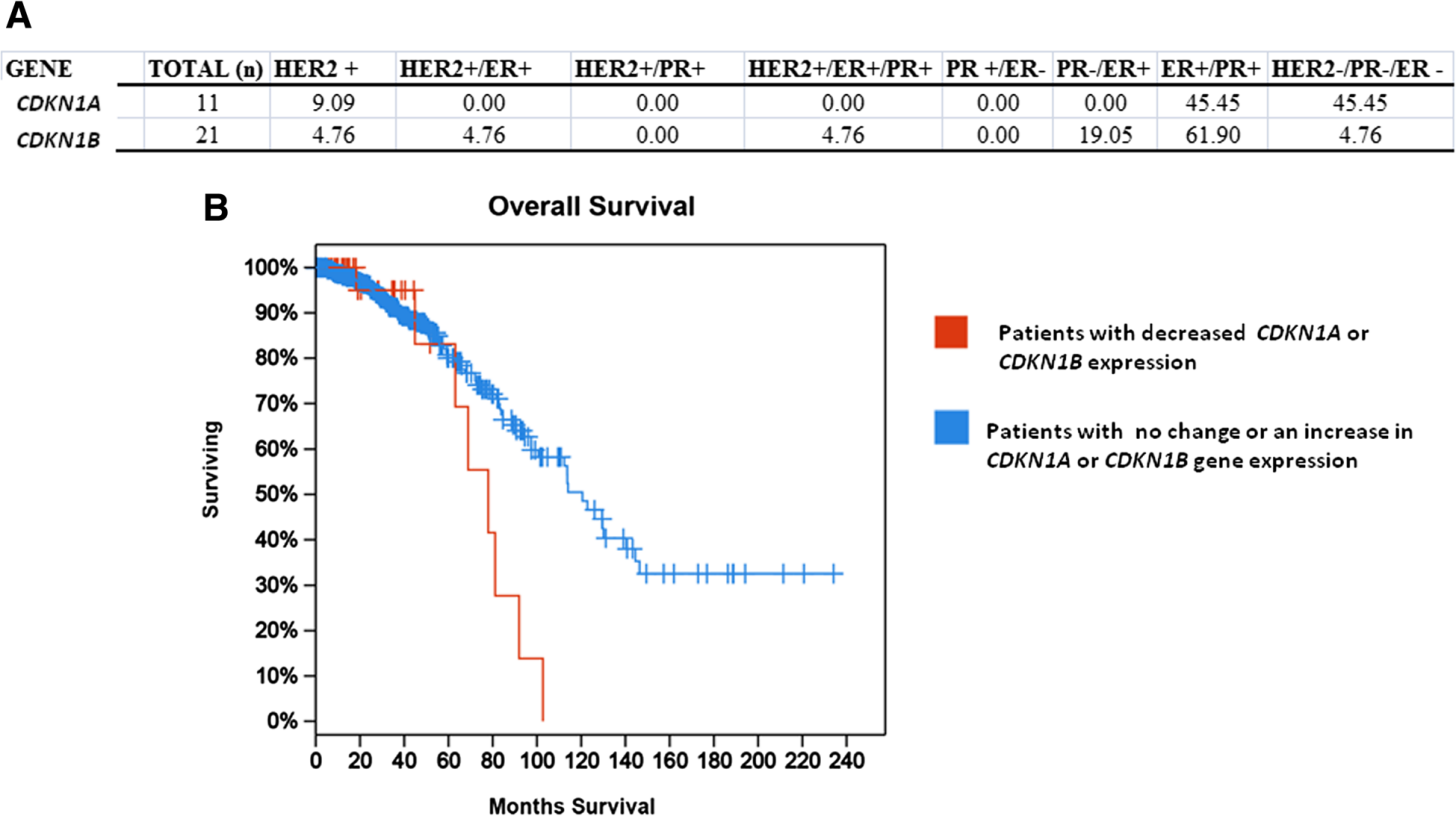
Patients from the TCGA Invasive Breast Carcinoma study [26, 27] with altered P21 and P27 had a tendency to be Her2 negative and had decreased survival. **a**. Estrogen receptor (ER), progesterone receptor (PR), and ErbB2 receptor (Her2) status in the 11 patients with decreases in the CDKN1a (P21) gene expression and ER, PR, and Her2 status of the 21 patients with decreases in the CDKN1b (P27) gene expression. **b**. Survival curve of patients with decreases in cdkn1a or cdkn1b gene expression compared to the 526 breast invasive carcinoma patients with mRNA Agilent microarray data. A rapid decrease in overall survival occurs in patients with decreases in cdnk1a or cdnk1b gene expression when compared to all patients with cancer

To further examine the connection of P21 and P27 expression in breast cancer, patients from the Cancer Genome Atlas (TCGA) invasive breast carcinoma cancer study were examined to determine whether expressions of Cdkn1a (P21) and Cdkn1b (P27) genes were inhibited in breast cancer tumors (TCGA, Nature 2012) [26, 27]. This data set contains 825 patients, of which data are available for 526 patients regarding mRNA expression levels from Agilent microarrays. Alterations in P21 or P27 expression occurred in 10 % of the 526 patients. Of those 52 patients, 56 % had decreases in P21, P27, or both. Four patients had decreases in both P21 and P27, 11 patients had decreases in P21, and 14 patients had decreases in P27. Of the 52 patients, information was available regarding human epidermal growth factor receptor 2 (Her2), estrogen receptor (ER), and progesterone receptor (PR) status in 32 patients. Interestingly, 45 % of patients with decreased expression of P21 were Her2, ER, and PR negative (Fig. 6a), and patients with lower levels of P21 or P27 had reduced survival times following diagnosis (Fig. 6b). This may suggest that cancer patients following an MR diet may benefit, since increased levels in P21 and P27 can inhibit the cancer cell proliferation, providing a longer period of time for other established cancer therapies to be effective.

## Discussion

Dietary MR has been identified as a strategy for disease prevention and increased lifespan in experimental animals [10, 19, 31, 32]. We hypothesize that MR may be used as a potential strategy for inhibiting carcinogenesis, and to test this hypothesis, we used the xenograft model for breast cancer by injecting MCF10AT1 cells into nude mice and examined the development tumors in these mice for 12 weeks. Our findings support this hypothesis and indicate that MR inhibits the growth of breast cancer tumors and induces apoptosis.

High levels of IGF1 and insulin in humans have been linked to increased risk of breast cancer [33]. In carcinogenesis, growth factors, such as IGF1 and insulin, stimulate growth and progression of cancer [34]. Therefore, the reduction in plasma IGF1 and insulin by MR suggest that the reduced levels in the insulin/IGF1 axis may inhibit tumor development in this xenograft model.

Methionine functions as the donor for the C2-C4 and amine nitrogen during the synthesis of the polyamines spermidine and spermine. Ornithine, the precursor of putrescine (which in turn is the precursor of spermidine and spermine), is increased in mice on the MR diet. Polyamines are involved in the regulation of proliferation, growth, and survival of cells [35]. High levels of polyamines have been identified in several cancers [36], and the inhibition of polyamine synthesis has been shown to have antitumor effects on skin, colon, and prostate cancers [37, 38]. The increase in ornithine suggests an inhibition of polyamine production. The metabolism of polyamines is strictly controlled and contains two rate-limiting enzymes: ornithine decarboxylase and S-adenosyl methionine decarboxylase [39]. Ornithine, spermidine, and spermine were previously reported to be increased in the liver of MR rats [29]. In the Perrone et al. 2012 study [29], gene expression of the two rate-limiting enzymes of polyamine synthesis in the liver were not inhibited. The conflicting findings regarding polyamine synthesis suggest that either MR effects on the tumor are independent of polyamine synthesis, or alternatively, polyamine function and synthesis are regulated differently in liver and mammary gland.

Taurine is a sulfur-containing amino acid that contributes to cell volume homeostasis and affects apoptosis mechanisms [40]. Taurine plasma levels are decreased in mice on MR. In cancer, volume-sensitive taurine correlates with cisplatin resistance [41]. Recently, taurine has been shown to induce apoptosis through PUMA (p53- up regulated modulator of apoptosis) in human colorectal, ovarian, and cervical cancer cells [41–43]. A decrease in taurine levels resulted in reduced cell volume that induced levels of activated caspase-3, which led to apoptosis in cervical adenocarcinoma cells [44]. Therefore, the MR effects on blood taurine levels (Table 2) could affect PUMA and lead to an increase in apoptosis in the mammary gland tissue and MCF10AT1 tumors of the MR mice, but this would need to be confirmed in future studies. Taurine has been examined as a novel tumor marker in female breast cancer patients. A decrease in serum taurine levels was observed in people with breast cancer or at high risk of breast cancer. Further, an inverse correlation between vascular endothelial growth factor (VEGF,marker for angiogenesis) and taurine concentrations has been demonstrated [45]. Therefore, the connection between taurine, MR, and cancer is convoluted and complicated, and further research is needed to understand the implication of reduced plasma levels of taurine in MR mice.

## Conclusion

In both the xenograft model and in breast cancer cell lines, the mechanism by which MR inhibits tumor progression appears to be a coordinated effort of inhibiting the cell cycle by stimulating the cell cycle inhibitors, P21 and P27. In both the MCF10AT1 tumors and surrounding mouse mammary tissue levels of P21 expression were elevated. Levels of P27 were not significantly changed in the xenograft model, suggesting that the effect of MR is more related directly to inhibiting P21 than P27. Both the metastatic breast cancer cell line (MDA-MB-231) and the immortalized breast cell line (MCF10A) responded to MR similarly to that seen in the xenograft model further supporting the relevance of P21 induction. In particular MDA-MB-231 cells had reduced cell proliferation after 24 h in MRCD media. Additionally, P27 also was elevated in the breast cancer cell lines, suggesting that it too may be involved in the suppression of tumor progression. Further evidence of P21 and P27 importance in patients with breast cancer was revealed in the analysis of the TCGA Breast Carcinoma Cancer study data set [26, 27]. Survival analysis revealed that patients with decreased P21 or P27 expression had reduced survival. This suggests that, if MR increases P21 and/or P27 expression, there may be an increased length of survival in breast cancer patients.

The results from the present study indicate that MR may be a protective agent against cancer progression, but does not completely inhibit cancer progression. Therefore, the application of MR in a clinical setting could be both safe and a feasible option for arresting the progression of breast cancer while providing patients with more time to be treated by conventional methods to eradicate the cancer. Further studies are needed to examine the effect of MR in combination with chemotherapeutic and immunotherapeutic treatments.

## Abbreviations

CC3, cleaved caspase-3; Cdkn1a, cyclin-dependent kinase inhibitor 1A (P21); Cdkn1b cyclin-dependent kinase inhibitor 1B (P27); CF, control fed; CR, caloric restriction; DAB, 3,3'-Diaminobenzidine; DCIS, ductal carcinoma *in situ*; ELISA, enzyme-linked immunosorbent assay; Her2, human epidermal growth factor receptor 2; IDPs, intraductal papillomas; MFP, mammary fat pad; MR, methionine restriction; MRCD, methionine-restricted, cysteine-depleted; NBF, neutral buffered formalin; PUMA, p53- up regulated modulator of apoptosis; ROS, reactive oxygen species; TC, total cholesterol; TCGA, the Cancer Genome Atlas; TG, plasma triglycerides; VEGF, vascular endothelial growth factor VEGF.

## Additional file

Additional file 1: Table S1. This file contains a table with the primer sequences used for RT-PCR on the mouse and human samples. (DOCX 13 kb)
