# Erratum to: Methionine-restricted diet inhibits growth of MCF10AT1-derived mammary tumors by increasing cell cycle inhibitors in athymic nude mice

**DOI:** 10.1186/s12885-016-2404-0

**Published:** 2016-07-14

**Authors:** J. R. Hens, I. Sinha, F. Perodin, T. Cooper, R. Sinha, J. Plummer, C. E. Perrone, D. Orentreich

**Affiliations:** Orentreich Foundation for the Advancement of Science, Inc., 855 Route 301, Cold Spring, NY 10516 USA; Biochemistry and Molecular Biology, Penn State College of Medicine, 500 University Drive, Hershey, PA 17033 USA; Comparative Medicine, Penn State College of Medicine, 500 University Drive, Hershey, PA 17033 USA

## Erratum

After publication of the original article [1], it was noticed that the uncorrected version of this article was published which contained a typographical error within Tables 1 and 2. Within Table 1 the value “11.4 ± 4 03” incorrectly read as “114 ± 4 03”. Within Table 2 the value “46.9 ± 17” incorrectly read as “46 9 ± 17”. These errors do not in any way effect the results, findings, interpretation, conclusions, or the scientific basis of the paper. The correct versions of Tables 1 and 2 can be found below, and have been corrected in the original article.

**Table 1:** Physiological parameters of mice on MR and CF diets. A 2-tailed t-test was conducted

	CF			MR		
	Mean (g) SD	Mean (%) SD	N	Mean SD	Mean (%) SD	N
Body weight	26.6 ± 2.79		18	23.9 ± 2.3***		20
Liver	1.13 ± 0.15	4.35 ± 0.43	19	0.988 ± 0.13 **	4.15 ± 0.49	20
Spleen	0.102 ± 0.02	0.383 ± 0.07	19	0.104 ± 0.15	0.436 ± 0.59	20
MFP (both)	0.271 ± 0.06	1.01 ± 0.20	18	0.275 ± 0.10	1.13 ± 0.35	20
Kidney (both)	0.343 ± 0.04	1.33 ± 0.19	19	0.303 ± 0.05 **	1.27 ± 0.19	20
Muscle-left side	0.123 ± 0.02	0.475 ± 0.89	19	0.081 ± 0.03 ****	0.339 ± 0.15 **	20
Tumor-wt (mg)	20.2 ± 6.1 ^a^	0.079 ± 0.30	15	11.4 ± 4 03 ^a^ ****	0.049 ± 0.02***	20

**** *p* ≤ 0.0001, *** *p* ≤ 0.001, ** *p* ≤ 0.01

^a^ tumor is in mg not g

**Table 2:** Plasma amino acid concentrations from mice on MR and CF diets. Levels of methionine, cysteine, and taurine in the plasma from mice on the MR diet are significantly lower than those on the control diet. The Kruskal-Wallis test was used to analyze the differences in amino acid concentration

	CF		MR	
	Mean (nmol/mL) SD	N	Mean (nmol/mL) SD	N
Ala	398 ± 85	17	520 ± 122 ***	18
Arg	133 ± 29	17	117 ± 21	18
Asp	11.8 ± 4.9	16	10.3 ± 3.1	18
Cys	4.39 ± 2.4	13	2.22 ± 0.44 *	9
Gln	622 ± 89	17	812 ± 106****	18
Glu	106 ± 20	17	117 ± 19	18
His	88.8 ± 11	17	112 ± 20***	18
Ile	94.8 ± 32	17	108 ± 27	18
Leu	139 ± 47	17	180 ± 46**	18
Met	248 ± 125	17	46.9 ± 17****	18
Orn	62.7 ± 29	17	114 ± 41***	18
Phe	114 ± 42	17	132 ± 39	18
Pro	69.5 ± 8.6	17	82.4 ± 34	18
Ser	218 ± 34	17	339 ± 84****	18
Tau	226 ± 50	17	77.1 ± 35****	18
Thr	346 ± 94	17	383 ± 136	18
Tyr	89.5 ± 19	17	107 ± 28*	18
Trp	130 ± 29	17	128 ± 29	18

**** *p* ≤ 0.0001, *** *p* ≤ 0.001, ** *p* ≤ 0.01, * *p* ≤ 0.05
