# Supplementary material for: Methionine-restricted diet inhibits growth of MCF10AT1-derived mammary tumors by increasing cell cycle inhibitors in athymic nude mice
Source: BMC Cancer. 2016 Jun 3;16:349. doi: 10.1186/s12885-016-2367-1 (PMC4891836; doi:10.1186/s12885-016-2367-1)
Supplement: Additional file 1: Table S1. — This file contains a table with the primer sequences used for RT-PCR on the mouse and human samples. (DOCX 13 kb) [file 12885_2016_2367_MOESM1_ESM.docx]

Supplementary Table S1

| **mRNA Accession #** | **Human Primers** | | | | **Sequence** |  |  |
| --- | --- | --- | --- | --- | --- | --- | --- |
|  | *HPRT* forward | | |  | 5'-CCTGGCGTCGTGATTAGTGA-3' | | |
| NM_000194.2 | *HPRT* reverse | | |  | 5'-CGAGCAAGACGTTCAGTCCT-3' | | |
|  | *CDKNA1* forward | | |  | 5'-GGCCCAGTGGACAGCGAGCA-3' | | |
| NM_000389 | *CDKNA1* reverse | | |  | 5'-CCCAGGCGAAGTCACCCTCC-3' | | |
|  | *CDKNB1* forward | | |  | 5'-TAACTCTGAGGACACGCATT-3' | | |
| NM_004064.3 | *CDKNB1* reverse | | |  | 5'-TGAGTAGAAGAATCGTCGGT-3' | | |
|  | *CCND1* forward | | | | 5'-AGACCTGCGCGCCCTCGGTG-3' | | |
| NM_053056.2 | *CCND1* reverse | | | | 5'-GTAGTAGGACAGGAAGTTGTTC-3' | | |
|  |  |  |  | |  |  |  |
|  | **Mouse Primers** | | | | **Sequence** |  |  |
|  | *Hprt* forward | |  | | 5'-GTTGGGCTTACCTCACTGCT-3' | | |
| NM_013556 | *Hprt* reverse | |  | | 5'-TCATCGCTAATCACGACGCT-3' | | |
|  | *Cdkna1* forward | |  | | 5'-CAACGCACCGAATAGTTACG-3' | | |
| NM_007669 | *Cdkna1* reverse | |  | | 5'-CAGCTCCTCAGCCAGGTC-3' | | |
|  | *Cdknb1* forward | |  | | 5'-AAGGGCCAACAGAACAGAAG-3' | | |
| NM_009875.4 | *Cdknb1* reverse | |  | | 5'-GGATGTCCATTCAATGGAGTC-3' | | |
|  | *Ccnd1* forward | | | | 5'-CACACGGACTACAGGGGAGT -3' | | |
| NM_007631.2 | *Ccnd1* reverse | | | | 5'-CACAGGAGCTGGTGTTCCAT-3' | | |
|  |  |  |  | |  |  |  |
